# Mn^2+^-Doped CsPbBr_2_I Quantum Dots Photosensitive Films for High-Performance Photodetectors

**DOI:** 10.3390/nano15060444

**Published:** 2025-03-15

**Authors:** Mengwei Chen, Wei Huang, Chenguang Shen, Yingping Yang, Jie Shen

**Affiliations:** 1 Department of Physics, School of Physics and Mechanics, Wuhan University of Technology, Wuhan 430070, China; mengwei.chen@whut.edu.cn (M.C.); huang17261767950@whut.edu.cn (W.H.); 347475@whut.edu.cn (C.S.); 2 State Key Laboratory of Advanced Technology for Materials Synthesis and Processing, School of Materials Science and Engineering, Wuhan University of Technology, Wuhan 430070, China; 3 Hubei Longzhong Laboratory, Wuhan University of Technology, Xiangyang Demonstration Zone, Xiangyang 441000, China

**Keywords:** Mn doping, CsPbBr_2_I quantum dots, photodetector, stability, photoresponse performance

## Abstract

The variable bandgap and high absorption coefficient of all-inorganic halide perovskite quantum dots (QDs), particularly CsPbBr_2_I make them highly promising for photodetector applications. However, their high defect density and poor stability limit their performance. To overcome these problems, Mn^2+^-doped CsPbBr_2_I QDs with varying concentrations were synthesized via the one-pot method in this work. By replacing Pb^2+^ ions, moderate Mn^2+^ doping caused lattice contraction and improved crystallinity. At the same time, Mn^2+^-doping effectively passivated surface defects, reducing the defect density by 33%, and suppressed non-radiative recombination, thereby improving photoluminescence (PL) intensity and carrier mobility. The optimized Mn:CsPbBr_2_I QDs-based photodetector exhibited superior performance, with a dark current of 1.19 × 10^−10^ A, a photocurrent of 1.29 × 10^−5^ A, a responsivity (*R*) of 0.83 A/W, a specific detectivity (D*) of 3.91 × 10^12^ Jones, an on/off ratio up to 10^5^, and the response time reduced to less than 10 ms, all outperforming undoped CsPbBr_2_I QDs devices. Stability tests demonstrated enhanced durability, retaining 80% of the initial photocurrent after 200 s of cycling (compared to 50% for undoped devices) and stable operation over 20 days. This work offers a workable strategy for rational doping and structural optimization in the construction of high-performance perovskite optoelectronic devices.

## 1. Introduction

In recent years, with the development of new quantum dot (QDs) materials and the innovation of device structure, quantum dot photodetectors have received extensive attention due to their unique advantages [1,2]. Among them, all-inorganic halide perovskite quantum dots (CsPbX_3_, X = Cl, Br, I) have excellent photoelectric properties such as high absorption coefficient, tunable emission spectrum, and so on [3,4,5]. It has become a research hotspot in the field of optoelectronics [3,6,7,8,9,10]. Compared with single halogen perovskite quantum dots, the mixed halogen perovskite quantum dots can achieve continuous adjustment of the band gap within a certain range by adjusting the halogen ratio to meet the needs of different optoelectronic applications, provide flexible adaptability for the design of optoelectronic devices, and become an ideal candidate material for optoelectronic devices [6,11,12]. However, although perovskite quantum dots have many advantages, their practical applications still face some challenges. First, even all-inorganic perovskites still have poor stability, especially under light, humidity, and high-temperature conditions, which can easily lead to performance degradation [13,14,15,16]. Secondly, it is easy to produce surface halogen vacancies and uncoordinated Pb^2+^, resulting in high defect state density, causing serious non-radiative recombination and significantly reducing photoluminescence (PL) quantum yield and carrier mobility [17]. The manipulation of material properties through doping is a fundamental aspect of device applications; therefore, how to improve the stability and photoelectric properties of perovskite quantum dots through material modification strategies has become one of the hotspots of current research [18,19,20,21].

Doping strategies have been widely explored to address the inherent limitations of perovskite materials. The high defect tolerance of the perovskite structure enables dopants to reduce defects introduced during the synthesis process and improve the orderliness of the lattice. The doping of transition metal ions (such as Mn^2+^) can not only adjust the band gap and optical properties of perovskite but also effectively passivate the surface defect states and relieve toxicity issues by reducing lead content, thereby improving the stability and photoelectric properties of the material [22,23,24]. Xu et al. synthesized Mn-doped CsPbCl_3_ nanocrystals, showing excellent PL properties and stability [25]. Liu et al. found that the stronger Mn-Cl and Pb-Cl bonds induced by Mn^2+^ doping enhanced the chemical stability of perovskite nanocrystals, and the lifetime of Mn^2+^-doped perovskite nanocrystals was extended by 10 times at a high acceleration voltage of 200 keV [26]. In addition, manganese doping has also been shown to improve the carrier lifetime and mobility of perovskite materials, which is of great significance for the performance improvement of photodetectors [27]. Although manganese doping has achieved remarkable progress in single halogen perovskites (such as CsPbCl_3_ and CsPbBr_3_) [28,29,30], the structure-activity relationship of doping concentration-structural stability/defect density-device performance in mixed halogen systems (such as CsPbBr_2_I) needs to be systematically revealed.

To address the aforementioned challenges of high defect-state density and poor stability in pure-phase CsPbBr_2_I QDs, this study developed a high-performance photodetector utilizing Mn^2+^-doped CsPbBr_2_I QDs. The Mn^2+^ doping strategy was implemented to modulate the crystal lattice structure and suppress non-radiative recombination, thereby enhancing both the optoelectronic properties and environmental stability of CsPbBr_2_I QDs. In this paper, Mn^2+^-doped CsPbBr_2_I QDs were prepared by a simple one-pot method. The effects of Mn^2+^ doping on the crystal structure and optical properties of CsPbBr_2_I QDs were systematically studied. A photodetector based on Mn:CsPbBr_2_I QDs was prepared, and its photoelectric properties and stability were evaluated. The results show that Mn^2+^ doping not only significantly improves the crystallinity and stability of quantum dots but also improves the responsivity, specific detectivity, on/off ratio, and response time of the photodetectors. This study provides new ideas and methods for the development of high-performance and stable perovskite quantum dot photodetectors.

## 2. Materials and Methods

### 2.1. Materials and Reagents

Cesium carbonate (Cs_2_CO_3_, 99.9%), lead bromide (PbBr_2_, 99.99%), 1-octadecene (ODE, >90%), oleylamine (OAm, 80–90%), trioctylphosphine (TOP, 90%), oleic acid (OA), methyl acetate (MeOAc, AR grade, ≥98%), and n-hexane (≥97%) were purchased from Shanghai Macklin Biochemical Co., Ltd., Shanghai, China. MnBr_2_ (98%) and titanium diisopropoxide bis(acetylacetonate) (75% in isopropanol) were purchased from Sigma-Aldrich (Shanghai) Trading Co., Ltd., Shanghai, China. Lead iodide (PbI_2_, 99.999%) was purchased from Xi’an Yuri Solar Co., Ltd., Xi’an, China. Octane (≥98%), N-hexane (≥97%), isopropanol (≥99.7%), acetone (≥99.5%), and absolute ethanol (99.7%) were purchased from China National Pharmaceutical Chemical Reagents Co., Ltd., Beijing, China. TiO_2_ slurry (30 NRD) was purchased from Yingkou Preferred Technology Co., Ltd., Yingkou, China. Carbon electrode paste (CH-8) was purchased from Ten Printing Equipment Technology (Pinghu) Co., Ltd. (JUJO Printing Supplies), Pinghu, China. Fluorine-doped tin oxide (FTO) was purchased from YouXuan Technology Co., Ltd., Shenzhen, China. All chemicals were used as received without further purification.

### 2.2. Synthesis and Purification of Mn:CsPbBr_2_I QDs

**Figure 1:**
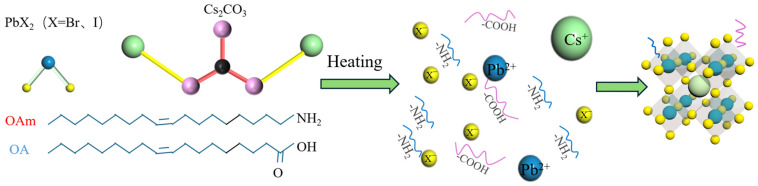
Reaction mechanism diagram of one-spot synthesis of CsPbBr_2_I QDs.

**Figure 2:**
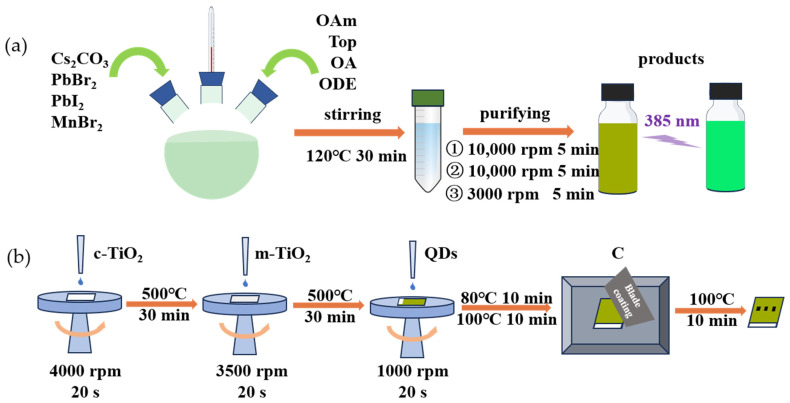
(**a**) Synthesis flow chart of Mn:CsPbBr_2_I QDs; (**b**) Preparation flow chart of photodetector.

Preparation of Mn:CsPbBr_2_I QDs: CsPbBr_2_I QDs were synthesized by the one-pot method; the reaction mechanism is shown in Figure 1, and the preparation process is shown in Figure 2a. First, 0.0675 mmol Cs_2_CO_3_, 0.2507 mmol PbBr_2,_ and 0.1254 mmol PbI_2_ were weighed in a nitrogen environment glove box and placed in a 50 mL three-necked round-bottom flask. In the air environment, the mixture was dissolved in 10 mL ODE, 1 mL OAm, 1 mL TOP, and 1 mL OA. Stir the mixture and heat it to 120 °C, stirring continuously for 30 min. The three-necked round-bottomed flask was cooled to room temperature in ice water to quickly stop the reaction. The preparation of Mn:CsPbBr_2_I QDs is the same as that of CsPbBr_2_I QDs. When the total molar amount of PbBr_2_ and MnBr_2_ remains unchanged, the molar ratio of MnBr_2_ to total Mn and Pb is about 16.7%, 33.3%, and 66.7%, respectively (the sample name is expressed in this ratio below). Other experimental materials remain unchanged, and the above preparation process is repeated.

Purification of Mn:CsPbBr_2_I QDs: An equal volume of methyl acetate was added to the reaction product, and the mixed solution was centrifuged at 10,000 rpm for 5 min to remove the supernatant. Add 5 mL n-hexane and 10 mL methyl acetate, and repeat the above experimental steps. Finally, 5 mL n-hexane was added and centrifuged at 3000 rpm for 5 min. The supernatant was taken and evaporated to dryness at 90 °C. The hexane and n-octane were added to the dried quantum dots to form 50 mg/mL, and the volume ratio of n-hexane to n-octane was 1:4 [31].

### 2.3. Fabrication of Photodetectors

The preparation process of the photodetector is shown in Figure 2b. The etched glass/FTO substrates were pre-cleaned by immersion in acetone, isopropanol, and anhydrous ethanol in an ultrasonic box, each followed by 30 min of ultrasonication. Take 0.1 mL diisopropyl alcohol diacetylacetone titanium and 1.9 mL ethanol, mix, and stir evenly. Preparation of mesoporous TiO_2_ (m-TiO_2_) slurry. Titanium dioxide and anhydrous ethanol with a mass ratio of 1:5 were mixed and stirred for 24 h.

The cleaned FTO substrate was dried and treated with ultraviolet ozone for 20 min. The c-TiO_2_ precursor solution was spin-coated on the FTO glass substrate; the rotation speed was 4000 rpm, the time was 20 s, and the annealing temperature was 150 °C for 10 min. The above operation was repeated once, and the final annealing temperature was 500 °C for 30 min. After cooling to room temperature, the preparation of c-TiO_2_ was completed. The m-TiO_2_ slurry was spin-coated on the dense layer at 3500 rpm for 20 s and annealed at 150 °C and 500 °C for 10 min and 30 min, respectively. After cooling to room temperature, the m-TiO_2_ layer was prepared and formed an electron transport layer with the dense TiO_2_ layer. The quantum dots dispersion was spin-coated on the electron transport layer at 1000 rpm for 20 s and repeated 3 times. The first two were annealed at 80 °C for 10 min, and the third was annealed at 100 °C for 10 min. Cooling to room temperature to form a Mn:CsPbBr_2_I QDs thin film photosensitive layer. Finally, the carbon counter electrode was prepared by screen printing and annealed at 100 °C for 10 min. At this point, the photodetector preparation is completed.

### 2.4. Characterization

The relative crystallinity, bulk phase structure, and cell changes after doping were characterized by X-ray diffraction (Empyrean, Malvern Panalytical, Malvern, UK), using Cu Ka radiation (λ = 1.54 Å). The atomic ratio of Mn to Pb in the Mn:CsPbBr_3_ QDs was measured by inductively coupled plasma-optical emission spectroscopy (ICP-OES) (Teledyne Leeman Labs, Prodigy7, Mason, OH, USA). The steady-state PL spectra were recorded by a fluorescence spectrometer (RF-6000, Shimadzu, Kyoto, Japan). An ultraviolet-visible spectrophotometer (UV-2600, Shimadzu, Kyoto, Japan) was used to measure the ultraviolet-visible (UV-Vis) absorption characteristics of quantum dot dispersions. The X-ray photoelectron spectroscopy (XPS) and band structure of quantum dots were measured by X-ray photoelectron spectroscopy (UPS, ESCALAB 250 Xi, Thermo Fisher Scientific, Waltham, MA, USA). Transmission electron microscopy (TEM) images, high-resolution transmission electron microscopy (HRTEM) images, Energy Dispersive Spectroscopy (EDS), and selected area electron diffraction (SAED) images of Mn:CsPbBr_2_I Ds were obtained by using 200 kV field emission high-resolution transmission electron microscopy (JEM-F200, JEOL, Tokyo, Japan). Among them, EDS quantitative analysis is based on the Cliff-Lorimer ratio model, which combines element characteristic X-ray intensity ratio with experimentally calibrated k-factor to achieve standardized quantitative calculation of element concentration in thin samples. The cross-sectional view of the photodetector was obtained by field emission scanning electron microscopy (SEM) (Zeiss Ultra Plus, Carl Zeiss, Oberkochen, Germany), and the acceleration voltage is 5000 V. The monochromatic adjustable light source (CME-OPS1000, Zhongke Micro Energy Technology Co., Ltd., Beijing, China) provides monochromatic light of different wavelengths and powers for the detector. The performance of the device was measured by a semiconductor parameter analyzer (4200A-SCS, Keithley, Cleveland, OH, USA). The electrochemical impedance spectroscopy (EIS) of the photodetector was obtained by electrochemical workstation (PP211, Zahner, Kronach, Germany) under AM 1.5 G radiation (irradiance of 100 mW/cm^2^) with alternating current (10 mHz to 10 MHz) at a bias of 0.7 V.

## 3. Results and Discussion

**Figure 3:**
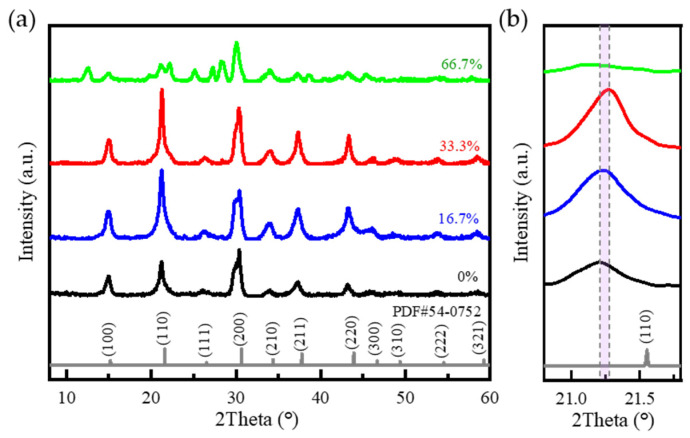
(**a**) XRD patterns of samples with different Mn doping ratios; (**b**) XRD enlarged patterns.

**Figure 4:**
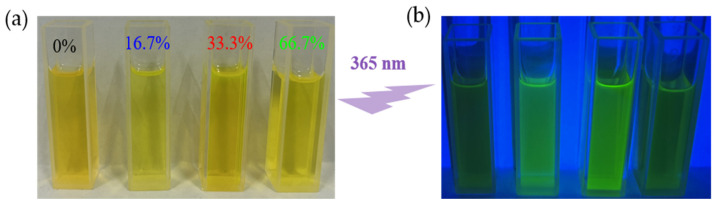
(**a**) Photos of the sample under ambient light; (**b**) Photos of the sample under 365 nm ultraviolet light excitationEDS tested the halogen ratio of 0% of the sample, and the atomic content of Br and I accounted for 65.26% and 34.74% of the total halogen content. The actual doping content of manganese was further tested using a full-spectrum direct-reading plasma emission spectrometer ICP-OES. The test results are shown in Table 2. In the 16.7% and 33.3% Mn-doped samples, the actual content of Mn accounted for 1.64% and 3.27% of the total content of Mn and Pb, respectively, which was positively correlated with the feed ratio. In the 66.7% Mn-doped sample, the actual content of Mn is only 0.3%, which is much lower than the feed ratio, indicating that excessive MnBr_2_ will lead to a decrease in the doping efficiency of Mn^2+^ in the lattice. The shift of the diffraction peak of the (110) crystal plane in XRD is highly consistent with the change of Mn content measured by ICP-OES, which further verifies that Mn is successfully doped into the perovskite lattice and reveals the significant effect of doping concentration on the crystal structure and optical properties. Appropriate Mn doping can effectively improve the crystal quality and optical properties of CsPbBr_2_I QDs, while excessive doping (66.7%) may lead to phase transition and doping efficiency reduction. This result provides an important basis for optimizing the Mn doping concentration and improving the photoelectric performance of perovskite quantum dots.

Mn:CsPbBr_2_I QDs with different manganese feed ratios (0%, 16.7%, 33.3%, 66.7%) were synthesized by the one-pot method, and their crystal structure and Mn doping content were systematically characterized by XRD and ICP-OES. Firstly, the crystal structure of the product was analyzed by XRD, and the test results are shown in Figure 3a. The XRD patterns of 0%, 16.7%, and 33.3% samples are consistent with the standard spectrum of CsPbBr_3_ (PDF # 54-0752) in terms of relative intensity and diffraction peak position, indicating that it has a cubic perovskite structure. Due to the introduction of halogen I, the position of the main peak (110) shifted to a low angle of 0.36°, confirming the successful synthesis of CsPbBr_2_I QDs. No other peaks were found in the XRD pattern, indicating that the sample had high purity. A total of 66.7% of the sample is due to the phase transition of the product caused by the excessive MnBr_2_ in the raw material. As shown in Figure 3b, with the increase in Mn doping concentration, the angle of the (110) crystal plane of Mn:CsPbBr_2_I QDs in the XRD spectrum moves higher than that of the host CsPbBr_2_I QDs. The sample 0% is 21.20°, the sample 16.7% is 21.23°, and the sample 33.3% is 21.27°. This is since the larger Pb^2+^ ion (about 1.2 Å) in the octahedral unit is slightly replaced by the smaller dopant Mn^2+^ ion (about 0.8 Å), resulting in lattice shrinkage, which proves that manganese is successfully combined into the perovskite lattice. Baseline fitting was performed on the original XRD pattern, and the valley bottom points of adjacent diffraction peaks were connected using linear interpolation to separate the crystalline peaks from the amorphous scattering background signal. Relative crystallinity (%) = 100 × (*A*_1_ − *A*_2_)/*A*_1_, where *A*_1_ is the total peak area after deducting the baseline, and *A*_2_ is the amorphous scattering area. As shown in Table 1, the relative crystallinity of the four samples was 77.26%, 79.38%, 99.04%, and 47.82%, respectively. The crystallinity of the 33.3% Mn-doped sample is the highest (99.04%), which is significantly better than that of the undoped sample (77.26%). It shows that an appropriate amount of Mn doping is helpful in improving the crystal quality, and the enhanced crystallinity is more conducive to the manufacture of photodetectors with high photoelectric performance and high reliability. As shown in Figure 4a,b, the Mn:CsPbBr_2_I QDs dispersed in n-hexane exhibit a yellow-green color under ambient light and emit bright green fluorescence under 365 nm UV excitation. Among them, the fluorescence intensity of 16.7% and 33.3% Mn-doped samples was significantly higher than that of other samples, indicating that appropriate Mn doping can effectively improve the luminescence performance of QDs. The fluorescence intensity was further quantitatively analyzed by PL test.

**Table 2:** The actual doping amount (atomic ratio) of Mn with different Mn/(Mn+Pb) feed ratios.

Mn/(Mn+Pb) feed ratios (at%)	16.7	33.3	66.7
The actual proportion of Mn (at%)	1.64	3.27	0.3

**Figure 5:**
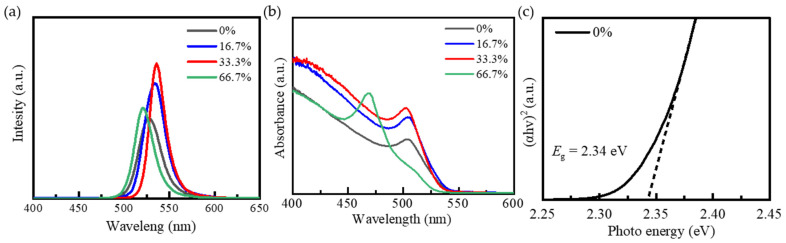
(**a**) PL spectra; (**b**) UV-Vis spectra; (**c**) Tauc plot of undoped CsPbBr_2_I QDs (0%).

To explore the effect of Mn doping on the optical properties of CsPbBr_2_I QDs, the photoluminescence (PL) spectra, absorption spectra, and band gap characteristics were systematically tested. The related PL peak position and FWHM data are shown in Table 3. Figure 5a is the photoluminescence spectra of CsPbBr_2_I QDs and Mn:CsPbBr_2_I QDs dispersed in n-octane. Undoped CsPbBr_2_I QDs show a narrow PL signal at 2.35 eV, corresponding to the radiation attenuation caused by band gap recombination. The appropriate amount of Mn doping significantly enhances the PL intensity, indicating that the introduction of Mn effectively inhibits non-radiative recombination and reduces the density of defect states (such as Pb vacancies), thereby improving the luminous efficiency of quantum dots. Compared with the undoped sample, the PL peaks of 16.7% and 33.3% of the samples were red-shifted, and the peaks were 2.32 eV and 2.31 eV, respectively. The PL peak of 66.7% samples blue-shifted to 2.38 eV, indicating that excessive MnBr_2_ in the synthesis may cause phase transition or defect state increase, resulting in band gap broadening. The PL full width at half maximum (FWHM) of the undoped, 16.7%, 33.3%, and 66.7% samples are 0.13 eV, 0.12 eV, 0.09 eV, and 0.11 eV, respectively. The 33.3% sample has the narrowest FWHM width (0.09 eV), which is consistent with the XRD results (crystallinity 99.04%), indicating that its crystal integrity is the best. Figure 5b is the absorption spectra of quantum dots with different Mn doping concentrations. All samples show similar absorption characteristics in the visible light range, and the absorption edge is located at ~530 nm, which corresponds to the band gap absorption of CsPbBr_2_I QDs. The 33.3% sample exhibits the strongest light absorption ability, which is attributed to its high crystallinity and low defect state density. The enhanced light capture ability is beneficial to the application of high-performance photodetectors. The optical band gap (*E*_g_) of undoped CsPbBr_2_I QDs (0%) is calculated by the Tauc plot method, as shown in Figure 5c; the band gap is 2.34 eV. Appropriate Mn doping can significantly improve the optical properties of CsPbBr_2_I QDs, which is manifested in the enhancement of PL intensity, the narrowing of half-peak width, and the improvement of light absorption ability. This result confirms the effectiveness of Mn^2+^ doping in inhibiting non-radiative recombination and optimizing band structure, which provides an important basis for the development of high-performance optoelectronic devices.

**Figure 6:**
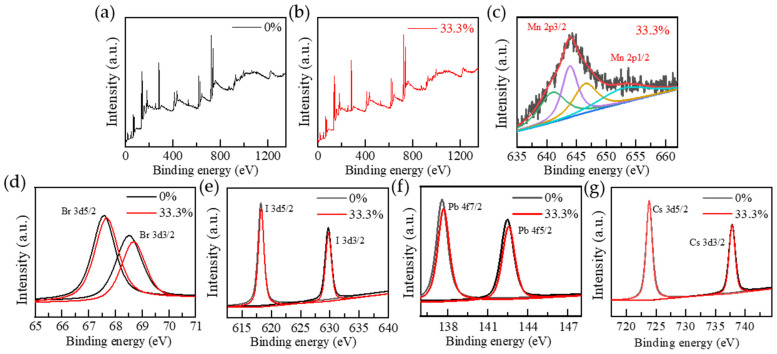
CsPbBr_2_I and Mn: CsPbBr_2_I QDs: (**a**,**b**) XPS spectra; (**c**–**g**) Core levels of Mn, Br, I, Pb, and Cs.

To explore the effect of Mn doping on the chemical state and coordination environment of CsPbBr_2_I QDs, XPS was used to systematically characterize the undoped (0%) and 33.3% doped samples. The test results are shown in Figure 6a,b. As shown in Figure 6c, the binding energies of Mn 2p3/2 and Mn 2p1/2 are located at 643.9 eV and 653.5 eV, respectively, which are consistent with the standard binding energy of Mn^2+^, confirming that Mn exists in quantum dots in the form of +2 valence. As shown in Figure 6d–g, the chemical states of Cs, Pb, Br, and I in 0% and 33.3% samples were confirmed by XPS data. The Cs 3d/2 and Cs 3d/2 junction energies of undoped and Mn-doped samples are 723.8 eV and 737.8 eV, respectively, and no obvious shift is observed, indicating that Mn^2+^ doping has little effect on the chemical environment of Cs. The Br 3d binding energy of CsPbBr_2_I QDs has two peaks, 67.58 eV and 68.50 eV, corresponding to Br 3d5/2 and Br 3d3/2, respectively. With the addition of Mn^2+^, the binding energy of Br 3d5/2 and Br 3d3/2 increased to 67.69 eV and 68.69 eV, respectively. Similar to the binding energy of Br, the two peaks of the binding energy of Pb 4f in the undoped sample are 137.59 eV and 142.48 eV, respectively, corresponding to Pb 4f7/2 and Pb 4f5/2, and the binding energy is also transferred to the high energy side after Mn^2+^ doping to 137.71 eV and 142.58 eV. Similar to Pb 4f, I 3d binding energy also showed two peaks: I 3d5/2 increased from 618.20 eV to 618.29 eV, and I 3d3/2 increased from 629.76 eV to 629.78 eV. Compared with the undoped samples, the high-resolution XPS peaks of Br, I, and Pb in the Mn^2+^-doped samples shifted slightly to higher binding energies, indicating that the coordination environments of Br, I, and Pb^2+^ changed slightly due to Mn^2+^ doping. The binding energy shift (Br 3d, I 3d, Pb 4f) caused by Mn^2+^ doping is mainly attributed to the change of coordination chemical environment caused by octahedral distortion. After Mn^2+^ (0.8 Å) replaced Pb^2+^ (1.2 Å), the lattice contraction and local stress increased, resulting in the redistribution of electron cloud density, which caused the binding energy to shift to the high energy side. In addition, no other impurity elements were detected by XPS full spectrum analysis, indicating that the sample had high chemical purity.

**Figure 7:**
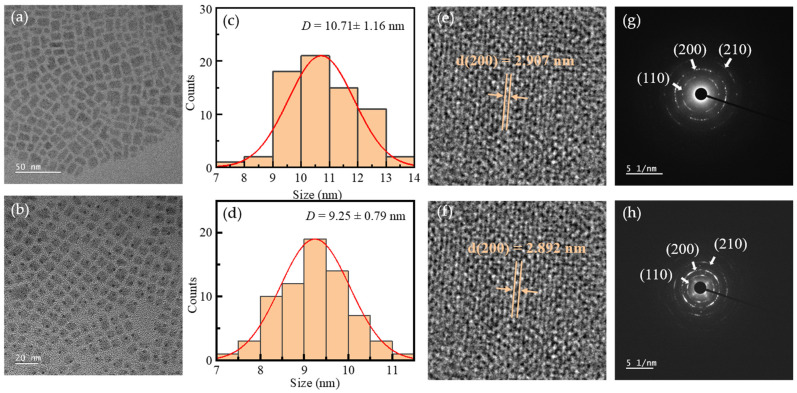
(**a**,**b**) TEM images, (**c**,**d**) Particle sizes distribution, (**e**,**f**) HRTEM images and (**g**,**h**) SAED patterns of CsPbBr_2_I and Mn:CsPbBr_2_I QDs, respectivel.

To further explore the effect of Mn^2+^ doping on the morphology and crystal structure of CsPbBr_2_I QDs, the undoped (0%) and 33.3% doped samples were systematically characterized by HRTEM. As shown in Figure 7a,b, the undoped and Mn^2+^-doped samples show a regular square morphology, which is consistent with the theoretical lattice structure of cubic perovskite. Figure 7c,d displays the histogram statistics of particle size distribution. The average particle sizes of the undoped and Mn^2+^-doped samples are 10.71 ± 1.16 nm and 9.25 ± 0.79 nm, respectively. After Mn^2+^ doping, the size distribution of quantum dots was more uniform, and the standard deviation decreased from 1.16 nm to 0.79 nm, indicating that Mn^2+^ doping effectively inhibited the non-uniform growth of quantum dots. The improvement of the size uniformity of quantum dots is because Mn^2+^ doping increases the defect formation energy and promotes the formation of a nearly uniform local structural order of perovskite quantum dots. The lattice contraction phenomenon of HRTEM and the binding energy shift of XPS confirmed the optimization effect of Mn^2+^ doping on the lattice coordination environment, which further supported the conclusion that the defect state density was reduced and the size uniformity was improved. The image shown in Figure 7e,f clearly shows the lattice fringes of quantum dots, indicating that they are highly crystalline. Figure 7g,h is the selected area electron diffraction pattern of the two samples. The doped samples exhibit more distinct diffraction rings, which aligns with the conclusion that the crystallinity of these samples is enhanced, as evidenced by the XRD analysis. Table 4 shows the interplanar spacing of two different quantum dots and the standard PDF (PDF # 54-0752). The (110), (200), and (210) interplanar spacings of undoped and Mn^2+^-doped samples are 4.143 Å, 2.907 Å, 2.579 Å, and 4.11 Å, 2.892 Å, 2.555 Å, respectively, which are highly consistent with the standard PDF (PDF # 54-0752). The interplanar spacings of (110), (200), and (210) decreased by 0.033 Å, 0.015 Å, and 0.024 Å, respectively, due to the substitution of smaller Mn^2+^ ions for larger Pb^2+^ ions, resulting in lattice contraction. The results of HRTEM reveal the key role of Mn^2+^ doping in optimizing the growth kinetics and local structural order of quantum dots, which provides an important basis for the development of high-performance optoelectronic devices.

**Figure 8:**
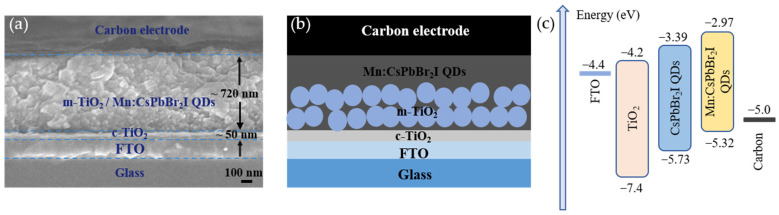
(**a**) Cross-section SEM image; (**b**) Device hierarchical structure diagram; (**c**) Energy level alignment diagram of the photodetector.

Based on the above characterization results, 33.3% Mn:CsPbBr_2_I QDs were selected as the photosensitive layer of the photodetector, and undoped CsPbBr_2_I QDs were used as a comparison sample to systematically study the effect of Mn^2+^ doping on device performance. The photodetector based on the Mn:CsPbBr_2_I QDs photosensitive layer is shown in Figure 8a as the cross-sectional SEM image, and Figure 8b is the structural schematic. The photodetector adopts a vertical structure, specifically a glass substrate/FTO/c-TiO_2_/m-TiO_2_/Mn:CsPbBr_2_I QDs photosensitive layer/carbon electrode. The thickness of the c-TiO_2_ electron transport layer is about 50 nm, and the total thickness of the m-TiO_2_/Mn:CsPbBr_2_I QDs photosensitive layer is about 720 nm. The m-TiO_2_ layer also plays the role of electron transport, and the porous structure provides a uniform filling space for the quantum dots, which enhances the light absorption and carrier transport efficiency. The energy band structure of Mn^2+^-doped CsPbBr_2_I QDs was investigated by UPS. As shown in Figure 8c, the conduction band minimum (CBM) and valence band maximum (VBM) of the undoped and Mn-doped samples were determined to be −3.39 eV/−5.73 eV and −2.97 eV/−5.32 eV, respectively, indicating an upward shift of energy levels induced by Mn^2+^ doping. Consequently, the energy offset (Δ*μ*) between the VBM of the photosensitive layer and the Fermi level (*E*_f_) of the carbon electrode (−5.0 eV) decreased from 0.73 eV (undoped) to 0.32 eV (Mn^2+^-doped), effectively reducing the energy-level mismatch at the photosensitive layer/carbon interface. This reduced energy barrier facilitates accelerated hole extraction and collection efficiency, as confirmed by the improved charge transport dynamics. These findings offer essential insights into band engineering strategies, guiding the design of high-performance perovskite optoelectronic devices with optimized interfacial energy alignment.

**Figure 9:**
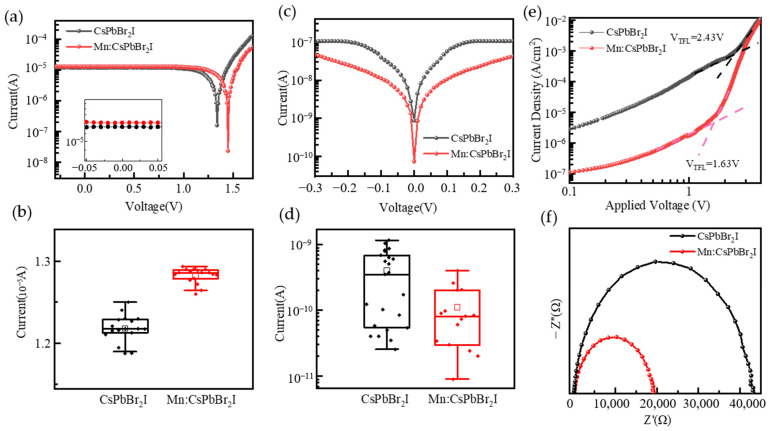
(**a**) Photocurrents and corresponding (**b**) box plots; (**c**) dark currents and corresponding (**d**) box plots; (**e**) SCLC test curves, (**f**) EIS curves of photodetectors based on CsPbBr_2_I and Mn:CsPbBr_2_I QDs films.

To systematically study the effect of Mn^2+^ doping on the performance of photodetectors, the photocurrent, dark current, defect state density, and interface charge transfer characteristics were tested and analyzed. From Figure 9a,b, it can be seen that the photocurrent of the device is 1.22 × 10^−5^ A and 1.29 × 10^−5^ A, respectively, and the dark current is 4.01 × 10^−10^ A and 1.19 × 10^−10^ A, respectively. Figure 9c,d show the corresponding box plots. The box plots of Mn^2+^-doped devices show smaller fluctuations in photocurrent and dark current, indicating higher repeatability and stability. Mn^2+^ doping increased the photocurrent by 5.7% while reducing the dark current by 70.3%, while the on/off ratio of the device reaches 10^5^, which is nearly an order of magnitude higher, indicating that Mn^2+^ doping significantly improved the optoelectronic performance of the device. The responsivity of the photodetector based on CsPbBr_2_I QDs is 0.79 A/W, and the specific detectivity is 2.01 × 10^12^ Jones. In contrast, the responsivity of the photodetector based on Mn:CsPbBr_2_I QDs is increased to 0.83 A/W, and the specific detectivity is increased to 3.91 × 10^12^ Jones. To further investigate the device performance, the Space charge limited current (SCLC) method was used to evaluate the defects of quantum dot thin films, as shown in Figure 9e. The well fill limit voltages (*V*_TFL_) of CsPbBr_2_I and Mn: CsPbBr_2_I quantum dot thin films were obtained through SCLC testing, which were 2.43 V and 1.63 V, respectively. Calculate the defect density of quantum dot thin films using the formula $N_{t}=2\varepsilon_{0}\varepsilon V_{\mathrm{TFL}}/(qL^{2})$, where $\varepsilon_{0}$ is the vacuum dielectric constant, *ε* is the relative dielectric constant of inorganic perovskite, *q* is the elementary charge, and *L* is the thickness of the perovskite films. According to the relationship between *N*_t_ and *V*_TFL_, after Mn^2+^ doping, the defect density of the film decreased by 33%. Mn^2+^ doping effectively reduces the defect density of quantum dots by alleviating lattice distortion and improving crystal integrity. This structural optimization promotes efficient carrier migration while suppressing non-radiative recombination processes. The above mechanism can explain the reduction in dark current and the increase in photocurrent in Mn^2+^-doped devices, ultimately achieving the synergistic optimization of photodetector responsivity and specific detection rate. By using EIS to study the charge transfer resistance at the device interface, the test results are shown in Figure 9f. The half-circle diameter of the EIS of Mn^2+^-doped devices is significantly smaller than that of undoped devices, indicating that Mn^2+^ doping effectively reduces the charge transfer resistance (*R*_ct_). This phenomenon is attributed to the optimization of the lattice structure of CsPbBr_2_I QDs by Mn^2+^ doping, which improves the efficiency of carrier separation and transmission.

**Figure 10:**
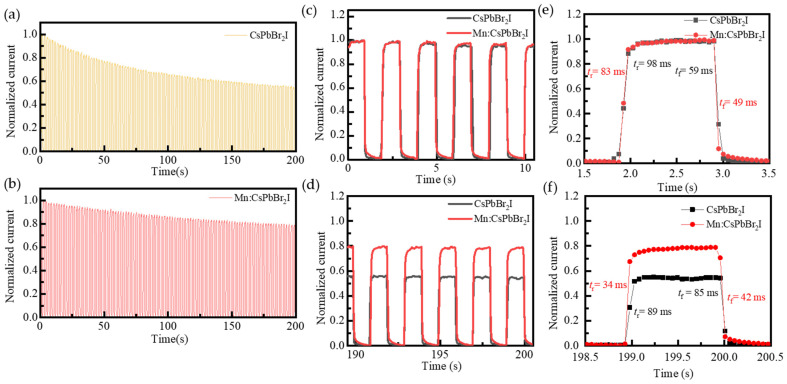
(**a**,**b**) Normalized optical on/off response characteristic curves of 200 s test cycle; (**c**,**d**) First and last 10 s detail diagrams; (**e**,**f**) Time response characteristic curves of photodetectors based on CsPbBr_2_I and Mn:CsPbBr_2_I QDs films.

Stability is a crucial performance metric for photodetectors, directly influencing their long-term reliability and measurement accuracy. To investigate the effect of Mn^2+^ doping on the stability of photodetectors, a photo responsive stability test was conducted on the device with a test period of 200 s. The test results are shown in Figure 10. Comparing the test results in Figure 10a,b, the photocurrent of Mn^2+^-doped devices showed higher stability during the 200 s test period, and their normalized photocurrent decay rate was significantly lower than that of undoped devices. Select 5 light on/off cycles before and after testing for comparison, as shown in Figure 10c,d. The Mn^2+^-doped device maintained 80% of its initial photocurrent after 200 s of testing, while the undoped device’s photocurrent decayed to 50% of its initial value. Mn^2+^ doping significantly reduces the non-radiative recombination rate by suppressing the generation of defect states, thereby delaying the decay of photocurrent. Figure 10e,f show the light on/off response characteristic curves for one cycle before and after the test. The rise time (*t*_r_) and fall time (*t*_f_) of Mn^2+^-doped devices are superior to those of the undoped devices. This result is attributed to the fact that Mn^2+^ doping reduces the interfacial charge transfer resistance (*R*_st_), enhances the carrier extraction efficiency, and thus shortens the response time. This further confirms the effectiveness of the Mn^2+^ doping strategy in improving the interface dynamics of CsPbBr_2_I QDs photodetectors.

**Figure 11:**
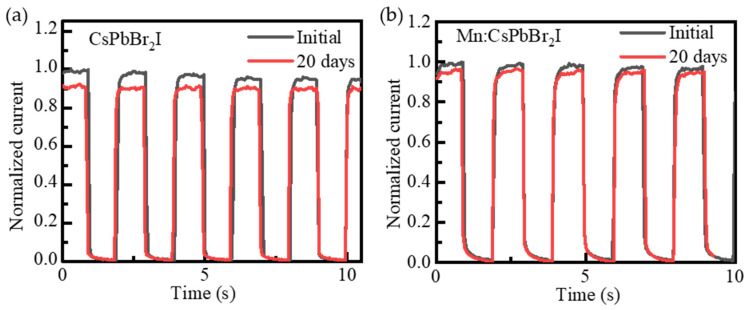
Long-term stability test of photodetectors based on (**a**) CsPbBr_2_I QDs films and (**b**) Mn:CsPbBr_2_I QDs films.

To explore the impact of Mn^2+^ doping on the long-term stability of the devices in environmental conditions, the optical switching response characteristics were measured for different quantum dot devices after a period of 20 days. Figure 11a and Figure 11b are the long-term stability test results of photodetectors based on CsPbBr_2_I QDs films and Mn:CsPbBr_2_I QDs films, respectively. The photocurrent of the Mn-doped device is 96% of the initial value, while that of the undoped device is only 89%. The transient photocurrent response stability within 20 days is significantly better than that of the device based on undoped QDs. The improvement of the environmental stability of Mn^2+^-doped CsPbBr_3_ QDs comes from the increase in formation energy and the passivation of defects after the substitution of Pb^2+^ by Mn^2+^ in perovskite crystals.

## 4. Conclusions

This study successfully achieved a synergistic improvement in the crystal structure, optical properties, and defect passivation of CsPbBr_2_I QDs through a controllable Mn^2+^ doping strategy. Mn^2+^ doping causes lattice shrinkage (reduction in interplanar spacing by ~0.8%) by replacing Pb^2+^ sites while reducing defect state density (33%) and improving crystal integrity (relative crystallinity increased from 77.26% to 99.04%). Narrowing the PL FWHM to 0.09 eV and significantly enhancing the PL intensity indicate effective suppression of non-radiative recombination. The absorption spectrum shows an improvement in light capture ability, consistent with the coordination field enhancement mechanism caused by lattice contraction. The photodetector based on the Mn: CsPbBr_2_I QDs photosensitive layer also achieved excellent performance. The photodetector exhibits low dark current (1.19 × 10^−10^ A), high photocurrent (1.29 × 10^−5^ A), and fast time response characteristics (rise/fall time optimization), responsivity, specific detectivity, and response time. The reduction in defect state density and improvement of carrier mobility (confirmed by SCLC and EIS) are the core mechanisms for performance optimization. The Mn^2+^-doped device retains 80% of the photocurrent after 200 s of optical switching cycles, which is much better than the undoped device (50%), and the long-term environmental stability (20 days) is significantly improved, attributed to the enhanced lattice stability and interface charge recombination suppression. However, further verification is still needed for long-term operation under extreme conditions (such as >60 °C or UV irradiation). This work reveals the regulation law of Mn^2+^ doping on the structure and properties of perovskite quantum dots, provides an example for defect engineering perovskite quantum dots, and offers new ideas for the development of high-reliability optoelectronic devices.

## Figures and Tables

**Table 1:** Relative crystallinity of the samples with different Mn/(Mn+Pb) feed ratios.

Mn/(Mn+Pb) feed ratios (at%)	0	16.7	33.3	66.7
Relative crystallinity (%)	77.26	79.38	99.04	47.82

**Table 3:** The PL peak position and FWHM of the samples with different Mn/(Mn+Pb) feed ratios.

Mn/(Mn+Pb) Feed Ratios	0%	16.7%	33.3%	66.7%
PL peak position (eV)	2.35	2.32	2.31	2.38
FWHM (eV)	0.13	0.12	0.09	0.11

**Table 4:** Interplanar spacing of samples 0%, 33.3%, and the standard PDF.

Samples	Diffraction Ring Diameter (nm^−1^)	Crystal Face	Interplanar Distance (Å)
PDF#54-0752	—	(110)	4.12
		(200)	2.915
		(210)	2.607
0%	4.827	(110)	4.143
	6.881	(200)	2.907
	7.756	(210)	2.579
33.3%	4.866	(110)	4.11
	6.915	(200)	2.892
	7.827	(210)	2.555

## Data Availability

Data are contained within the article.
